# Exploring the Role of Novel Colour‐Changing Toothpaste as an Aid for Improving Children’s Oral Hygiene Behaviour: An In Vivo Study

**DOI:** 10.1155/ijod/1011514

**Published:** 2026-04-01

**Authors:** Nithya Annie Thomas, Rebecca Ann Simon, Subramaniam Ramanarayanan, Charisma Thimmaiah, Shweta Kajjari

**Affiliations:** ^1^ Department of Pediatric and Preventive Dentistry, Manipal College of Dental Sciences, Manipal Academy of Higher Education, Manipal, 576 104, Karnataka, India, manipal.edu,; ^2^ Manipal College of Dental Sciences, Manipal Academy of Higher Education, Manipal, 576 104, Karnataka, India, manipal.edu; ^3^ Department of Public Health Dentistry, Indira Gandhi Institute of Dental Sciences, Nellikuzhi, Kothamangalam, 686691, Kerala, India, igids.ac.in; ^4^ Manipal College of Dental Sciences Mangalore, Manipal Academy of Higher Education, Manipal, India, manipal.edu; ^5^ Department of Pediatric and Preventive Dentistry, KLE V.K. Institute of Dental Sciences, KLE Academy of Higher Education and Research, Belagavi, 590010, Karnataka, India

**Keywords:** motivation, oral hygiene, paediatric dentistry, plaque index, toothpaste

## Abstract

**Background:**

Driving children to maintain good oral hygiene prevails as a universal concern. Colour‐changing toothpastes render visual feedback designed to enhance brushing technique, plaque elimination and enthusiasm.

**Aim:**

To assess the efficacy of an innovative colour‐changing toothpaste in enhancing brushing performance, motivation and plaque scores among children aged 6–12 years.

**Methodology:**

A parallel arm randomised controlled trial was conducted amid 100 children aged 6–12 years. Study subjects were assigned into an experimental group (colour‐changing toothpaste) and a control group (standard non‐colour‐changing toothpaste) using block randomisation. Brushing technique instruction was standardised. Plaque index (Silness and Loe) was documented at baseline (P0), 5 weeks (P1) and 10 weeks (P2). Motivation and behaviour were evaluated utilising validated child and parent‐based Likert scales and daily brushing logs.

**Results:**

The results showed that the colour‐changing toothpaste depicted slow yet consistent enhancement in plaque reduction, oral hygiene habits, brushing technique awareness and motivation, with significant results across most comparisons. However, parental perception improved significantly in the conventional group, whereas parental satisfaction was higher and more consistent in the colour‐changing toothpaste post‐intervention.

**Conclusion:**

The integration of visual indicators in toothbrushing may contribute to a noteworthy role in boosting oral hygiene behaviour among children.

**Trial Registration:** Clinical Trials Registry of India: CTRI/2025/08/092295

## 1. Introduction

‘Good habits formed in childhood make all the difference’.—Aristotle.

Setting effectual oral hygiene practices through childhood is obligatory to intercepting dental caries and periodontal problems subsequently in life. Nevertheless, various hurdles‐like inadequate motivation, mediocre brushing technique and dearth of supervision—add to suboptimal oral hygiene behaviours among children [1–3]. Traditional toothbrushing practices may fail to engage children, making compliance inconsistent.

Innovative visual feedback based dental hygiene tools, like colour‐changing toothpastes and plaque indicator dyes, bestow an optimistic approach to considerably augmenting brushing calibre by furnishing prompt cues concerning brushing potency or duration [4–9]. Such response aids children in noticing areas skipped during brushing and/or enhances their brushing span and technique [8, 9]. Colour‐changing dentifrices, a novel behavioural reinforcement tool, are devised to provide children with real time visual feedback. These formulations alter colour after around 2 min of toothbrushing, thereby steering children on brushing duration and surging the pleasure of the task [7, 10]. This change in colour helps children to identify that the brushing time is complete. The rationale behind it is that it visually highlights the time needed to complete toothbrushing. By incorporating a fun, economical and interactive feature into the oral hygiene routine, the colour‐changing toothpaste could increase children’s engagement, making brushing a more exciting and rewarding experience [7, 10, 11]. Earlier studies have revealed that dye containing or plaque indicating dentifrices can remarkably enhance the plaque removal and brushing compliance compared to conventional formulations [1, 2, 5, 6].

Despite these advantages, current scientific evidence on the role of such toothpastes remains sparse. Earlier investigations have studied plant‐based, fluoridated, natural and desensitising compositions, yet very limited studies have assessed visual feedback toothpastes particularly for brushing habits, motivation and plaque outcomes [12–14]. Moreover, child cognitive development favours visual and interactive learning, supporting the theoretical benefit of visual aids in behaviour modification [15–18]. This research aims to tackle this breach by assessing the efficacy of a food grade and risk‐free colour‐changing toothpaste compared with an established non‐colour altering toothpaste in enhancing children’s oral hygiene behaviour over a 10‐week period. The objectives of the study were to examine whether the colour‐changing toothpaste improves children’s brushing technique, plaque removal and a long term oral hygiene habits; to assess whether the visual feedback provided enhances motivation and enjoyment during brushing; to measure the changes in brushing technique and brushing frequency overtime; to compare the effectiveness of the colour‐changing toothpaste with that of standard toothpaste; to collect feedback from participants regarding their overall brushing experience. The expected outcomes of the study suggested better brushing techniques, with children using the colour‐changing toothpaste anticipated to demonstrate better plaque removal; increased motivation, as the colour‐changing feature is expected to make brushing more engaging and encourage consistent brushing; enhanced long term oral hygiene, with the experimental group presumed to maintain healthier habits overtime, ultimately leading to better oral health outcomes. It is anticipated that the results of this study will provide insightful information on how children’s behaviour can be changed and their oral health improved using non‐invasive, entertaining and commonplace technologies.

## 2. Materials and Methods

This study was designed as a prospective and parallel arm randomised controlled trial that was carried out in a school in Karnataka’s Udupi District over a period of 10 weeks. The institutional ethics committee gave their approval (IEC1: 266/2025). Prior to recruitment, parents or guardians of every child participating in the study provided written informed consent. Based on inclusion and exclusion criteria, 100 healthy children without any systemic ailments between the ages of 6 and 12 who could brush their teeth on their own were chosen. Children undergoing orthodontic therapy, systemic disorders or a history of toothpaste ingredient allergies were excluded.

Sample size estimation was carried out assuming a standard deviation of 0.6, an alpha error of 1%, a study power of 95% and a clinically significant difference of 0.36, the required sample size was calculated to be 50 participants in each group, resulting in a total of 100 children. Statistical significance was set at *p*  < 0.05  for all comparisons.

Participants were randomly allocated into two groups using block randomisation with a block size of four to maintain equal distribution across the intervention arms. Concealment of allocation was ensured using sequentially numbered, sealed and opaque envelopes. The experimental group (G1) was provided with an innovative and novel colour‐changing toothpaste (Clove Kidz Toothpaste), and the control group (G2) received a conventional non‐colour‐changing children’s toothpaste. Both dentifrices complied with regulatory standards and were provided to the study participants along with standardised toothbrushes. All the participants were instructed to use the assigned toothpaste twice daily, for a period of 10 weeks. The modified Bass toothbrushing technique was demonstrated to all participants at baseline and at 5 weeks to ensure uniformity in brushing practices. The experimental group’s (G1) colour‐changing toothpaste includes safe and food‐grade pigments that, when brushed, combine to give a purple colour, indicating around 2 min of brushing. Two safe and food‐grade natural colourants were carefully combined to produce the colour shift that was seen. These colours combine to create a vivid purple glow when activated by water and brushing motion, making oral hygiene more engaging and encouraging children to brush consistently. The control toothpaste (G2) contained standard components such as sorbitol, silica, SLS, sodium monofluorophosphate and stabilisers, but lacked any colour‐changing properties. Outcome assessments were carried out at three time points: Baseline (P0), mid‐intervention at 5 weeks (P1) and post‐intervention at 10 weeks (P2). During each evaluation session, a calibrated expert recorded the Silness and Loe Plaque index for each participant, which was used to assess plaque accumulation as the primary clinical outcome. The assessment of the primary outcome was performed by a blinded examiner who was unaware of the group allocation of the participants. Secondary outcome measures included motivation for brushing, enjoyment of brushing, frequency of brushing and length of brushing. Validated Likert scale questionnaires designed specifically for parents and children were used to evaluate these. To record brushing frequency, length and adherence to the prescribed toothpaste, parents also kept daily brushing diaries. The questionnaires assessed the oral hygiene practices, brushing behaviour, motivation levels and parental perception. Table 1 shows the list of codes and their description

**Table 1:** List of codes and description.

Code	Description
H1	How many times does your child brush in a day?
H2	How long does your child usually brush his/her teeth?
H3	Does your child brush independently or require supervision?
H4	How often does your child miss toothbrushing sessions?
B1	Does your child know the correct method of toothbrushing?
B2	Does your child brush all the areas of the mouth (front teeth and back teeth)?
M1	I enjoy brushing my teeth
M2	Brushing teeth is boring
M3	I feel happy after brushing
M4	I think brushing teeth is important
M5	I feel like a hero after brushing my teeth
P1	How would you rate your child’s interest in brushing teeth?
P2	Have you used any methods/tools to motivate your child’s brushing habit?
A1	Did you like using the colour‐changing toothpaste?
A2	Was it fun to see the toothpaste changing its colour while brushing?
A3	Did the colour change help you to understand when to finish brushing?
A4	Did you feel more excited to brush your teeth because of the colour‐changing toothpaste?
A5	Will you continue to use the colour‐changing toothpaste in future?
A6	Did you brush your teeth for a longer time because of the colour‐changing toothpaste?
L1	Did you notice any changes in your child’s brushing behaviour after using the colour‐changing toothpaste?
L2	Did the colour change make it easier to monitor your child’s brushing?
L3	Did your child seem more motivated to brush because of the colour‐changing toothpaste?
MB	Morning brushing
EB	Evening brushing
UB	Toothpaste usage

All collected data were analysed using SPSS software version 23.0. Normality of data distribution was verified using the Shapiro–Wilk test. Paired *t*‐tests were used for intragroup comparisons across time points for normally distributed variables, whereas independent *t*‐tests were used for intergroup comparisons. The Mann–Whitney *U* test and Wilcoxon signed‐rank test were used appropriately for variables that were not regularly distributed. *p* less than 0.05 was chosen as the threshold for statistical significance.

## 3. Results

A total of 100 children completed the study, with 50 participants each in Group 1 (conventional toothpaste) and Group 2 (colour‐changing toothpaste).

### 3.1. Plaque Index Scores

Table 2 presents the comparison of plaque index scores between the two groups at baseline (P0), Week 5 (P5) and Week 10 (P10). At baseline, there was no significant difference between the plaque scores between the groups (*p* = 0.056). By Week 5, Group 1 exhibited significantly lower plaque scores compared to Group 2 (*p*  < 0.001). At Week 10, plaque scores remained significantly higher in Group 2 than in Group 1 (*p* = 0.014).

**Table 2:** Comparison of plaque index scores between the groups at baseline (P0), Week 5 (P5) and Week 10 (P10).

Time	Group	Count	Mean (SD)	Median (interquartile range)	Range	Mann–Whitney *U*
P0	Group 1	50	0.82 (0.425)	0.77 (0.54, 1.08)	0.08–1.7	*p* = 0.056
	Group 2	50	0.963 (0.394)	0.93 (0.7, 1.2)	0.08–1.9	
P5	Group 1	50	0.536 (0.575)	0.43 (0.2, 0.67)	0–3.7	*p* < 0.001 ^∗^
	Group 2	50	0.891 (0.511)	0.905 (0.45, 1.33)	0–1.83	
P10	Group 1	50	0.075 (0.15)	0 (0, 0.12)	0–0.83	*p* = 0.014 ^∗^
	Group 2	50	0.224 (0.31)	0.04 (0, 0.33)	0–1.29	

*Note:* Group 1: conventional toothpaste; Group 2: colour‐changing toothpaste.

^∗^Significant at *p*  < 0.05.

### 3.2. Oral Hygiene Habits, Brushing Technique Awareness, Motivation and Enjoyment, Parental Perception and Tooth Brushing Log Parameters

Table 3 describes intergroup comparison of oral hygiene habits, brushing technique awareness, motivation and enjoyment, parental perception and tooth brushing log parameters.

**Table 3:** Intergroup comparisons of oral hygiene habit parameters (H1–H4).

Time	Group	Count	Mean (SD)	Median (interquartile range)	Range	Mann–Whitney *U*
H1	Group 1	29	1.931 (0.258)	2 (2, 2)	1–2	*p* = 0.0125
	Group 2	34	1.794 (0.41)	2 (2, 2)	1–2	
H2	Group 1	29	2.241 (0.511)	2 (2, 2)	2–4	*p* = 0.352
	Group 2	34	2.324 (0.475)	2 (2, 3)	2–3	
H3	Group 1	29	1 (0)	1 (1, 1)	1–1	*p* = 1.000
	Group 2	34	1 (0)	1 (1, 1)	1–1	
H4	Group 1	29	1.655 (0.936)	1 (1, 2)	1–4	*p* = 0.137
	Group 2	34	1.294 (0.462)	1 (1, 2)	1–2	
B1	Group 1	29	1.103 (0.31)	1 (1, 1)	1–2	*p* = 0.057
	Group 2	34	1 (0)	1 (1, 1)	1–1	
B2	Group 1	29	1.552 (0.506)	2 (1, 2)	1–2	*p* = 0.001
	Group 2	34	1.147 (0.359)	1 (1, 1)	1–2	
M1	Group 1	29	1.138 (0.351)	1 (1, 1)	1–2	*p* = 0.07
	Group 2	33	1.364 (0.549)	1 (1, 2)	1–3	
M2	Group 1	29	2.483 (0.829)	3 (2, 3)	1–3	*p* = 0.221
	Group 2	34	2.706 (0.676)	3 (3, 3)	1–3	
M3	Group 1	30	1.333 (0.479)	1 (1, 2)	1–2	*p* = 0.293
	Group 2	34	1.235 (0.496)	1 (1, 1)	1–3	
M4	Group 1	29	1.069 (0.258)	1 (1, 1)	1–2	*p* = 0.489
	Group 2	34	1.059 (0.343)	1 (1, 1)	1–3	
M5	Group 1	29	1.483 (0.574)	1 (1, 2)	1–3	*p* = 0.141
	Group 2	34	1.676 (0.535)	2 (1, 2)	1–3	
P1	Group 1	28	1.571 (0.634)	1.5 (1, 2)	1–3	*p* = 0.873
	Group 2	34	1.529 (0.563)	1.5 (1, 2)	1–3	
P2	Group 1	17	2.647 (0.786)	3 (2, 3)	1–4	*p* = 0.938
	Group 2	6	2.667 (0.516)	3 (2, 3)	2–3	
MB	Group 1	31	67.194 (8.979)	70 (70, 70)	28–70	—
	Group 2	36	65.833 (12.54)	70 (67, 70)	12–70	
EB	Group 1	31	65.129 (10.95)	70 (66, 70)	27–70	—
	Group 2	36	63.083 (16.89)	69 (67, 70)	0–70	
UT	Group 1	30	66.783 (9.79)	70 (67, 70)	28–74	—
	Group 2	36	64.944 (12.64)	70 (67, 70)	12–70	

*Note:* Group 1: conventional toothpaste; Group 2: colour‐changing toothpaste.

^∗^Significant at *p*  < 0.05.

Intergroup comparisons of oral hygiene habit parameters (H1–H4) showed no statistically significant differences between the groups for any of the habit‐related variables (*p*  > 0.05).

Regarding brushing technique awareness parameters (B1 and B2), B1 showed a trend toward higher scores in Group 1 (*p* = 0.057) and B2 demonstrated significantly higher scores in Group 1 compared to Group 2 (*p* = 0.001), reflecting better awareness of brushing technique in this domain.

Across all motivation and enjoyment parameters (M1–M5), no statistically significant intergroup differences were observed. Group 2 consistently showed higher median values across several motivation domains; however, these did not reach statistical significance (*p*  > 0.05).

Parental perception parameters (P1 and P2) showed comparable scores between the groups, with no statistically significant differences observed.

The comparison of toothbrushing log parameters reveals that morning brushing (MB), evening brushing (EB), and toothpaste usage (UT) frequencies were similar between the two groups, and none of the differences were statistically significant (*p*  > 0.05).

### 3.3. Post‐Intervention Child and Parent Questionnaires

Intergroup comparisons of post‐intervention child (A1–A6) and parent (L1–L3) questionnaire responses are presented in Table 4. No statistically significant differences were observed between the groups for any of the parameters.

**Table 4:** Intergroup comparisons of post‐intervention child and parent questionnaire responses.

Time	Group	Count	Mean (SD)	Median (interquartile range)	Range	Mann–Whitney *U*
A1	Group 1	27	1.185 (0.483)	1 (1, 1)	1–3	*p* = 0.944
	Group 2	34	1.147 (0.359)	1 (1, 1)	1–2	
A2	Group 1	27	1.519 (0.58)	1 (1, 2)	1–3	*p* = 0.117
	Group 2	34	1.294 (0.462)	1 (1, 2)	1–2	
A3	Group 1	27	1.148 (0.456)	1 (1, 1)	1–3	*p* = 0.768
	Group 2	34	1.118 (0.409)	1 (1, 1)	1–3	
A4	Group 1	27	1.296 (0.542)	1 (1, 2)	1–3	*p* = 0.187
	Group 2	34	1.441 (0.504)	1 (1, 2)	1–2	
A5	Group 1	27	1.296 (0.542)	1 (1, 2)	1–3	*p* = 0.824
	Group 2	34	1.265 (0.511)	1 (1, 1)	1–3	
A6	Group 1	27	1.407 (0.694)	1 (1, 2)	1–3	*p* = 0.627
	Group 2	34	1.441 (0.613)	1 (1, 2)	1–3	
L1	Group 1	27	1.148 (0.362)	1 (1, 1)	1–2	*p* = 0.006 ^∗^
	Group 2	33	1.515 (0.566)	1 (1, 2)	1–3	
L2	Group 1	26	1.308 (0.471)	1 (1, 2)	1–2	*p* = 0.037 ^∗^
	Group 2	33	1.606 (0.556)	2 (1, 2)	1–3	
L3	Group 1	26	1.308 (0.618)	1 (1, 1)	1–3	*p* = 0.751
	Group 2	33	1.212 (0.415)	1 (1, 1)	1–2	

*Note:* Group 1: conventional toothpaste; Group 2: colour‐changing toothpaste.

^∗^Significant at *p*  < 0.05.

Regarding the post‐intervention parent questionnaire Group 2 demonstrated significantly higher scores for L1 (*p* = 0.006) and L2 (*p* = 0.037) compared to Group 1, indicating a more favourable parental perception toward the colour‐changing toothpaste. No significant difference was observed for L3.

Table 5 shows the intragroup analysis of all the parameters. It is observed that both groups experienced a significant decrease in their plaque index scores throughout the study duration. The conventional toothpaste group achieved significant plaque reduction at every time‐point comparison. The colour‐changing toothpaste group showed significant improvement between P0–P10 and P5–P10, indicating a more gradual yet sustained plaque reduction pattern. The two groups showed major enhancements in oral hygiene habits (H1–H4), yet the colour‐changing toothpaste group maintained significant results throughout all pairwise comparisons, and the conventional group showed non‐significant results in certain comparisons. The two groups showed major improvements in their brushing technique awareness (B1 and B2). The colour‐changing toothpaste group showed consistent positive changes in motivation and enjoyment towards brushing (M1–M5), but the conventional group showed only particular non‐significant changes. The conventional toothpaste group showed major improvements in parental perception (P1 and P2), but the colour‐changing toothpaste group showed only a minor non‐significant increase. The post‐intervention child questionnaire (A1–A6) showed meaningful progress in both groups, but the colour‐changing toothpaste group maintained more consistent results. The post‐intervention parent questionnaire (L1–L3) showed that only L3 scores improved significantly in the colour‐changing toothpaste group. This group showed better parental satisfaction after the intervention.

**Table 5:** Intragroup comparison of the parameters using *t* test.

Parameter	Comparison	Conventional toothpaste *p* value	Colour‐changing toothpaste *p* value
Plaque index scores— Baseline (P0), Week 5 (P5) and Week 10 (P10)	P0 with P5	<0.001 ^∗^	0.409
	P0 with P10	<0.001 ^∗^	<0.001 ^∗^
	P5 with P10	<0.001 ^∗^	<0.001 ^∗^
Oral hygiene habit parameters (H1–H4)	H1 with H2	0.017 ^∗^	<0.001 ^∗^
	H1 with H3	<0.001 ^∗^	<0.001 ^∗^
	H1 with H4	0.133	<0.001 ^∗^
	H2 with H3	<0.001 ^∗^	<0.001 ^∗^
	H2 with H4	0.001 ^∗^	<0.001 ^∗^
	H3 with H4	0.001 ^∗^	0.001 ^∗^
Brushing technique awareness	B1 with B2	0.001 ^∗^	0.023 ^∗^
Motivation and enjoyment towards brushing (M1–M5)	M1 with M2	<0.001 ^∗^	<0.001 ^∗^
	M1 with M3	0.023 ^∗^	0.211
	M1 with M4	0.161	0.001 ^∗^
	M1 with M5	0.002 ^∗^	0.006 ^∗^
	M2 with M3	<0.001 ^∗^	<0.001 ^∗^
	M2 with M4	<0.001 ^∗^	<0.001 ^∗^
	M2 with M5	<0.001 ^∗^	<0.001 ^∗^
	M3 with M4	0.006 ^∗^	0.012 ^∗^
	M3 with M5	0.096	<0.001 ^∗^
	M4 with M5	0.001 ^∗^	<0.001 ^∗^
Parental perception (P1 and P2)	P1 with P2	<0.001 ^∗^	0.034
Post‐intervention child questionnaire responses (A1–A6)	A1 with A2	0.001 ^∗^	0.169
	A1 with A3	0.327	0.711
	A1 with A4	0.185	0.001 ^∗^
	A1 with A5	0.185	0.325
	A1 with A6	0.083	0.039 ^∗^
	A2 with A3	0.001 ^∗^	0.136
	A2 with A4	0.031 ^∗^	0.096
	A2 with A5	0.110	0.831
	A2 with A6	0.449	0.169
	A3 with A4	0.043 ^∗^	0.006 ^∗^
	A3 with A5	0.043 ^∗^	0.169
	A3 with A6	0.032 ^∗^	0.009 ^∗^
	A4 with A5	1.00	0.184
	A4 with A6	0.327	1.000
	A5 with A6	0.449	0.205
Post‐intervention parent questionnaire responses (L1–L3)	L1 with L2	0.212	0.263
	L1 with L3	0.161	0.001 ^∗^
	L2 with L3	1.000	<0.001 ^∗^

^∗^Significant at *p*  < 0.05.

## 4. Discussion

Visual feedback aids can fortify optimal brushing duration and refine children’s enthusiasm—aspects perceived to regulate cumulative plaque levels [4–9, 13, 17]. The present study assessed the effectiveness of a colour‐changing toothpaste in improving brushing behaviour, plaque reduction and brushing technique in children. Both groups demonstrated significant improvement overtime; however, children using colour‐changing toothpaste showed distinct advantages, particularly in early plaque reduction and brushing technique awareness [7, 10, 17, 18].

Comparison with prior studies shows that while several toothpaste formulations have been evaluated for plaque control and gingival health [7, 10, 17], very limited evidence exists regarding colour‐changing formulations. This study is expected to contribute novel insights into behavioural aspects, compliance, and oral hygiene improvement through interactive brushing aids. Consistent with earlier studies on dye containing and plaque disclosing toothpastes, the colour‐changing group exhibited significantly lower plaque scores at the 5‐ and 10‐week intervals [1, 2, 5, 6]. This supports the evidence that visual cues enhance plaque removal by guiding children to brush for an adequate duration and cover all tooth surfaces more effectively [4, 8, 9]. Visual feedback tools—whether disclosing agents, colour‐changing toothpastes or interactive toothbrushes—have repeatedly been shown to improve brushing accuracy and increase brushing time [18, 19].

The superior brushing technique awareness observed in the colour‐changing toothpaste group further highlights the role of visual learning in behavioural change. Studies have shown that children respond strongly to visual pedagogy, with significant improvements brushing technique and oral hygiene skills when feedback tools are incorporated [18, 20]. The real time colour changes likely served as a behavioural reinforcement cue, encouraging children to adopt and maintain correct brushing techniques [7, 10, 17].

Interestingly, although both groups improved in motivation scores, there were no significant intergroup differences. This may indicate that supervised brushing programmes themselves can positively influence children’s interest and motivation, as reported in previous trials [10, 11, 21].

However, the colour‐changing toothpaste appeared to accelerate early behavioural adaptation, reinforcing the notion that interactive tools can facilitate faster engagement in younger populations [7, 10, 18].

Parental perception scores were higher in the control group. Previous research suggests that parents may perceive visible behavioural changes—such as reduced plaque build‐up and improved brushing independence—differently depending on expectations and familiarity with the product used [15, 16, 21]. Nonetheless both the groups improved overall, indicating that structured oral hygiene reinforcement benefits children.

Overall, the findings of the current study propose that colour‐changing toothpaste is a potent adjunct in paediatric oral hygiene. It enhances early plaque reduction, refines brushing technique and furnishes a visually engaging experience that may help children incorporate favourable brushing routines. These outcomes are consistent with contemporary behaviour modification models, which emphasise visual feedback, reinforcement learning and child friendly tools to improve long term oral hygiene practices [17, 18]. Colour‐changing toothpaste may, therefore, serve as a practical, low cost and enjoyable tool in both home based, and school based preventive dentistry programmes [20, 22–24]. Irrespective of the dentifrice used, supervised toothbrushing and consistent behavioural reinforcement prevail as the backbone of effective oral hygiene maintenance in children. Adult supervision guarantees correct brushing technique, appropriate duration and satisfactory plaque removal, while positive reinforcement and continual guidance help manifest habit formation and skill maintenance. Children who are under regular supervision and motivation demonstrate significantly better plaque control and oral hygiene outcomes than those who brush independently, underscoring that adjunctive products are most effective when integrated into organised and supervised oral health education programmes rather than used in isolation.

## 5. Conclusion

Colour‐changing toothpaste displays a heralding, engaging and child focussed practice to upgrade oral hygiene habits. Compared to traditional toothpaste, this interactive component boosts motivation and improves compliance. In the end, these oral behaviour‐enhancing tools can significantly aid in the development of proper brushing practices and effective long‐term proficient long term oral health benefits.

## Funding

This work was partially supported by the Star Dental Centre Private Limited and MedEd Foundation, Bangalore.

## Conflicts of Interest

The authors declare no conflicts of interest.

## Data Availability

No new data were generated or analysed in this study. All data supporting the findings of this study are available within the article.
